# From Contouring to Rejuvenation: A Nationwide Big-Data Analysis of Hyaluronic Acid Injection Trends in Japan

**DOI:** 10.3390/jcm15020893

**Published:** 2026-01-22

**Authors:** Taichi Tamura, Takahiko Tamura, Kohki Okumura, Hiroo Teranishi

**Affiliations:** Tokyo Chuo Beauty Clinic Umeda Osaka Ekimae, Osaka 530-0057, Japan

**Keywords:** hyaluronic acid injection, aging and rejuvenation, injection site trends, big data analysis, facial rejuvenation trend

## Abstract

**Background**: Hyaluronic acid (HA) injections have become a cornerstone of minimally invasive aesthetic medicine. While the demand for these procedures continues to grow globally, large-scale longitudinal analyses of patient demographics and specific injection site trends remain limited, particularly in Asian populations. Existing data in Japan are largely confined to aggregate procedure numbers. This study aimed to elucidate the transition in patient demographics and site-specific treatment trends using a nationwide big-data approach. **Methods**: This retrospective study analyzed 299,413 treatment sessions (417,590 injection sites) from patients who underwent facial HA injections at 110 clinics across Japan between October 2020 and December 2024. Data were analyzed by year, patient age, and injection site to evaluate demographic shifts and treatment patterns. **Results:** The annual number of treatment sessions increased steadily during the study period. A significant demographic shift was observed: while patients in their 20s were predominant in 2020–2022, the proportion of patients aged ≥ 40 years increased markedly from 2023 onward, accounting for more than half of all cases (63.7% in 2024). Treatment preferences varied distinctly by age; younger patients favored localized contouring (e.g., pretarsal fullness, chin), whereas older patients required multi-site rejuvenation. By 2024, the orbital rim became the most frequently treated site (22.6%). Statistical analysis confirmed that age was a significant predictor for multi-site treatments (*p* < 0.001). **Conclusions**: This large-scale analysis reveals a clear transition in the Japanese aesthetic market from contour enhancement in younger demographics to anatomy-based rejuvenation in middle-aged and older populations.

## 1. Introduction

Hyaluronic acid (HA) injections are widely performed worldwide as minimally invasive procedures for facial contouring and rejuvenation. Owing to their immediate effects, versatility, and favorable safety profile, facial HA injections have become one of the most commonly performed nonsurgical cosmetic treatments globally.

The growing demand for these procedures is well-documented in Western countries. According to the 2024 procedural statistics from the American Society of Plastic Surgeons (ASPS), over 5.3 million hyaluronic acid filler procedures were performed in the United States, representing a 1% increase from the previous year and marking it as the second most popular minimally invasive procedure after neuromodulator injections [1]. International data from the International Society of Aesthetic Plastic Surgery (ISAPS) Global Survey corroborate this trend, reporting 6.3 million HA filler procedures worldwide in 2023, representing a 5.2% increase from 2022 [2]. Notably, a 14-year longitudinal analysis by ISAPS revealed a 40% overall increase in aesthetic procedures over the past four years alone, underscoring the sustained global growth of this market [2].

These large-scale registries provide valuable insights into overall procedural volume and basic age stratification. The ASPS data reveal that individuals aged 40–54 years constitute the largest patient cohort (approximately 46% in 2023), and when combined with those aged 55 and older, mature patients account for the vast majority of the market [1]. Interestingly, while Western registries document stable or increasing demand among middle-aged and older populations, recent European studies have identified a concurrent rise in aesthetic procedures among younger adults. A multicenter analysis from the Netherlands demonstrated that patients aged 18–25 years represented a growing proportion of HA filler recipients, with their share increasing from 3.1% in 2008 to 8.0% in 2017 [3]. This divergence in age-related trends between different regions highlights the need for population-specific analyses.

However, while these registries offer robust data on total procedural volume and age stratification, they lack granular information regarding specific injection sites and treatment complexity. Consequently, it remains unclear from aggregate numbers whether the high prevalence of procedures among older cohorts reflects simple volume replacement in a single area or complex, multi-site rejuvenation strategies addressing the multifactorial nature of facial aging.

Recent clinical evidence suggests that the demographic profile of patients seeking aesthetic medical care is evolving. In a previous nationwide analysis, we demonstrated a significant increase in middle-aged and older individuals visiting cosmetic surgery clinics in Japan, accompanied by a parallel rise in lower eyelid transconjunctival fat removal procedures [4]. This trend reflects a growing demand among older patients for treatments addressing age-related facial changes, particularly in the periorbital region. While surgical data provide insight into definitive interventions, HA injections offer a broader “barometer” of aesthetic trends as they are applicable across all age groups for diverse indications.

Despite the growing market, detailed epidemiological data on HA injections stratified by specific anatomical sites remain scarce. Furthermore, ethnic differences in facial aging and aesthetic ideals necessitate population-specific analyses. East Asian faces are characterized by distinct skeletal and soft tissue features compared to Caucasians, including greater midfacial retrusion, wider zygoma, and thicker skin [5,6]. These anatomical differences influence not only the aging process but also treatment approaches and aesthetic goals. Importantly, facial aesthetic treatments in Asian populations are not aimed at “Westernization” but rather reflect culture-specific beauty standards and address ethnicity-specific aging patterns [6,7].

The present study aimed to elucidate recent changes in facial HA injection patterns in Japan using a nationwide multiclinic database. By analyzing over 417,000 injection sites across nearly 300,000 treatment sessions, we sought to characterize the transition in patient demographics and injection site preferences, and to bridge the gap in epidemiological knowledge regarding site-specific treatment trends in an Asian population.

## 2. Materials and Methods

This retrospective cohort study was approved by the local ethics committee of Tokyo Chuo Beauty Clinic (TCB) (Approval No. UMEDAERB-2025Jun002). The requirement for written informed consent was waived due to the retrospective nature of the analysis. The study was conducted in accordance with the ethical standards outlined in the Declaration of Helsinki.

We evaluated a comprehensive database of facial HA injections performed at TCB clinics nationwide between October 2020 and December 2024. The TCB network comprises clinics located in major cities across all 47 prefectures of Japan. During the study period, the number of participating clinics increased from approximately 100 facilities in 2021 to 110 facilities by the end of 2024. The database included all patients who underwent facial HA injections during this period, ensuring complete representation of clinical practice within the group.

A total of 299,413 treatment sessions, corresponding to 417,590 injection sites, were included in the final analysis. It is important to note that the term “patients” throughout this manuscript refers to treatment sessions (i.e., clinic visits), not unique individuals. Each visit by the same patient was counted separately, representing a distinct treatment decision. Similarly, when multiple anatomical sites were treated during a single session, each site was recorded as an independent injection event. Therefore, the reported “patient count” represents the total number of treatment sessions, and data on unique patient counts (distinguishing repeat visitors from first-time patients) were not available in this retrospective database.

In 2021, the clinic network transitioned to a standardized product line (YVOIRE, LG Chem, Iksan-si, Republic of Korea) and implemented a unified anatomical site classification system. This transition prompted the establishment of a consistent electronic medical record (EMR) system with standardized data entry protocols. To ensure consistency in site-specific analyses, data from 2020 (2231 sessions; 2597 sites) were excluded from all injection-site distribution and site-specific logistic regression analyses. However, age-related demographic trends were analyzed across the full study period (2020–2024) because patient age was recorded consistently throughout.

The YVOIRE portfolio encompasses a range of formulations with varying viscoelastic properties suitable for diverse anatomical applications, from superficial (Classic) to deep structural volumization (Volume and Contour). All procedures followed a standardized workflow: anatomical assessment and site selection were performed first, after which the treating physician selected the most appropriate YVOIRE formulation based on the tissue characteristics of the chosen site. This sequence ensures that product availability did not dictate injection site selection, and that clinical indications remained the primary driver of treatment decisions.

All injection sites were recorded using a standardized EMR system with a predefined dropdown menu implemented across the clinic network in 2021. The classification system comprised 14 anatomical regions based on recognized landmarks and functional aesthetic units (Supplementary Table S1): forehead, temple, above eye (retro-orbicularis oculi fat [ROOF] region), orbital rim (tear trough), pretarsal fullness (aegyo-sal), mid-cheek groove (nasojugal groove/Golgo line), nose, doll cheek, cheek, nasolabial fold (NLF), lip, marionette line, chin, and others.

Each treatment session was recorded by the treating physician immediately after the procedure, with each site documented as a separate entry when multiple sites were treated during a single session. 

Data extracted from the EMR system included patient age at the time of treatment, specific injection site(s), and the number of sites treated per session. For the purpose of analysis, patient age was categorized into six groups: <20 years, 20–29 years, 30–39 years, 40–49 years, 50–59 years, and ≥60 years. Safety data regarding serious complications were also reviewed from the database records and have been reported separately [8].

Descriptive statistics were used to assess temporal trends and distribution patterns. Annual patient volume, age distribution, and injection site frequencies were calculated and visualized. To evaluate age-dependent treatment preferences, heat map analyses were performed showing the proportion of specific injection sites across different age groups.

Logistic regression analyses were conducted to examine two primary outcomes: (1) the likelihood of undergoing multi-site treatment (≥2 injection sites during a single session), and (2) the likelihood of receiving injections at specific anatomical sites. For both analyses, binary logistic regression models were constructed with age category as the independent variable, using the “under 20 years” group as the reference category (reference odds ratio = 1.0). The models did not adjust for year of treatment or clinic-level clustering, as the primary objective was to characterize age-related treatment patterns rather than to control for temporal or institutional effects.

Odds ratios (OR) with 95% confidence intervals (CI) were calculated to quantify the magnitude of association between age and treatment patterns. A two-tailed *p*-value of <0.05 was considered statistically significant. All statistical analyses were performed using Stata version 19.5 (StataCorp LLC., College Station, TX, USA).

During the preparation of this manuscript, the authors used Genspark AI (Genspark, version accessed January 2026, https://www.genspark.ai) for manuscript editing and literature search assistance. Specifically, the AI tool was utilized to: (1) refine and proofread the English language text for grammar, clarity, and academic writing style; (2) search and identify relevant peer-reviewed literature, including recent publications from ASPS, ISAPS, and academic databases; and (3) assist in organizing references in the required citation format. All AI-generated suggestions were critically reviewed, verified, and edited by the authors to ensure accuracy and appropriateness. The AI tool was not used for study design, data collection, statistical analysis, data interpretation, or generation of scientific conclusions. All scientific content, data analysis, and interpretations remain the sole responsibility of the authors.

## 3. Results

**Figure 1:**
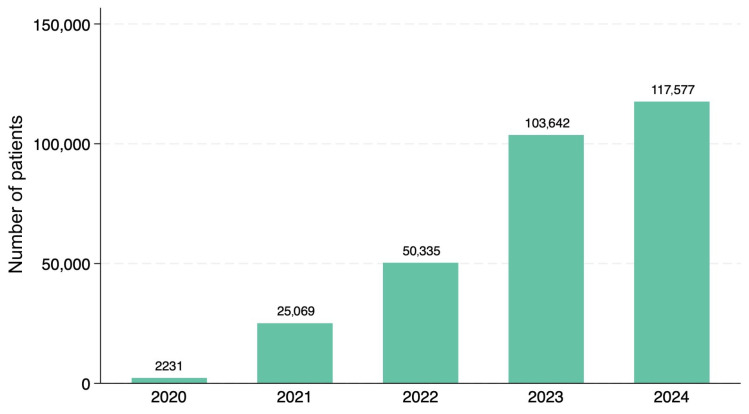
Annual number of treatment sessions from 2020 to 2024. The total number of sessions increased steadily each year, reaching 117,577 in 2024. This growth was accompanied by a gradual demographic shift toward middle-aged and older individuals. Note: 2020 represents a partial year (October–December only).

The total number of treatment sessions increased steadily over the study period. Table 1 summarizes the annual clinic count, treatment sessions, injection sites, and key demographic indicators. The clinic network expanded from 64 facilities in 2021 to 110 facilities in 2024. Notably, while clinic expansion contributed to overall growth, the mean number of patients per clinic increased substantially from 391.7 in 2021 to 1068.9 in 2024, indicating genuine market growth beyond network expansion alone. The mean number of injection sites per session also increased from 1.32 in 2021 to 1.50 in 2024, reflecting growing treatment complexity (Figure 1).

**Figure 2:**
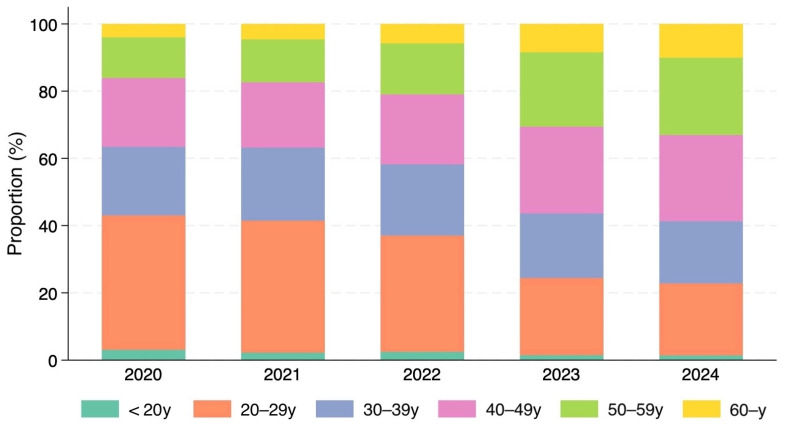
Annual distribution of patient age groups from 2020 to 2024. In 2020 (October–December), patients in their 20s accounted for the largest proportion of cases. However, this proportion decreased annually as the share of patients aged ≥ 40 years increased. By 2023, patients in their 40s became the largest demographic group, and from 2023 onward, individuals aged 40 years and older accounted for more than half of all treated cases.

This growth was accompanied by a significant demographic shift in the patient population. In 2020, patients in their 20s accounted for the largest proportion of cases. However, this demographic distribution changed annually, with the proportion of patients aged 40 years and older increasing progressively (Figure 2).

By 2023, patients in their 40s had become the largest age group, and from 2023 onward, individuals aged 40 years and older accounted for more than half of all treated cases, reaching 63.7% in 2024 (Table 1).

**Figure 3:**
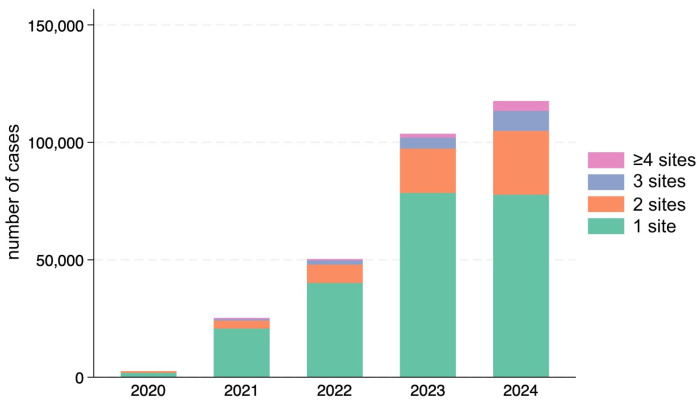
Annual distribution of patients categorized by the number of treated facial sites per session. The proportion of patients receiving multi-site injections (≥2 regions) increased consistently each year. Based on visual analysis and calculation from mean sites per session (Table 1), approximately 40% of sessions in 2024 involved multiple sites, compared to approximately 32% in 2021. This trend correlates with the rising proportion of middle-aged and older patients, who were found to be significantly more likely to undergo injections at multiple sites compared to younger individuals (*p* < 0.001).

Parallel to this shift in age, the complexity of treatments also increased. The likelihood of receiving injections at multiple facial sites during a single session was found to increase significantly with age (*p* < 0.001). Consequently, the overall proportion of patients receiving injections at two or more sites rose each year, reflecting the growing population of middle-aged and older patients requiring comprehensive correction rather than single-site treatment (Figure 3).

**Figure 4:**
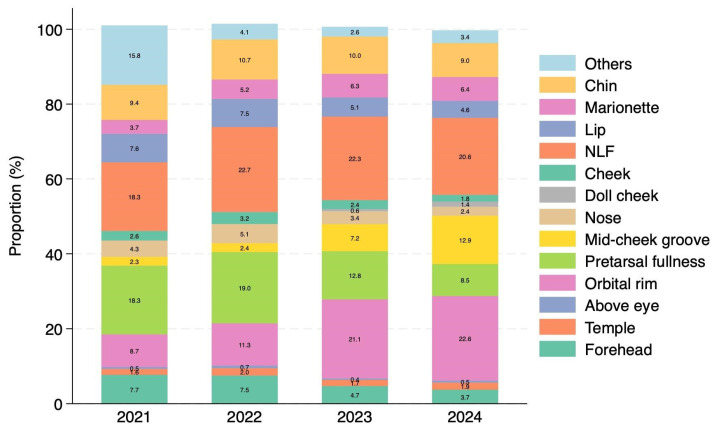
Annual changes in the percentage distribution of HA injection sites from 2021 to 2024. Data from 2020 were excluded due to non-standardized site classification prior to 2021. In the earlier years (2021–2022), the nasolabial folds (NLF) and pretarsal fullness were the most frequently treated sites. A significant shift was observed over the study period, with orbital rim injections increasing steadily to become the most common treatment site by 2024 (22.6%).

The distribution of specific treatment sites also evolved markedly. In 2021 and 2022, the nasolabial folds and pretarsal fullness were the most frequently treated areas. However, injections to the orbital rim and glabella saw a steady increase, and by 2024, the orbital rim had become the most common treatment site, accounting for 22.6% of procedures, followed by the nasolabial folds at 20.6% (Figure 4).

**Figure 5:**
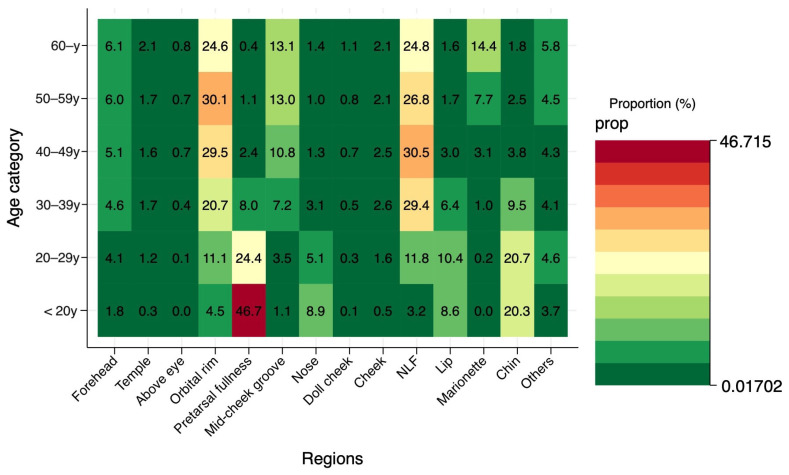
Proportion of specific injection sites within each age category. A clear age-dependent divergence in treatment goals is observed. Among patients in their teens and 20s, pretarsal fullness and chin augmentation were the most common procedures, reflecting a preference for contour enhancement. Conversely, in patients aged 50 and older, the orbital rim and nasolabial folds became predominant, while pretarsal fullness injections decreased to negligible levels, reflecting a shift toward rejuvenation.

Detailed analysis of injection sites by age group revealed distinct treatment preferences. Proportional analysis showed that among patients under 20 years of age, pretarsal fullness (46.7%) and chin injections were predominant, reflecting a primary desire for aesthetic contouring (Figure 5).

**Figure 6:**
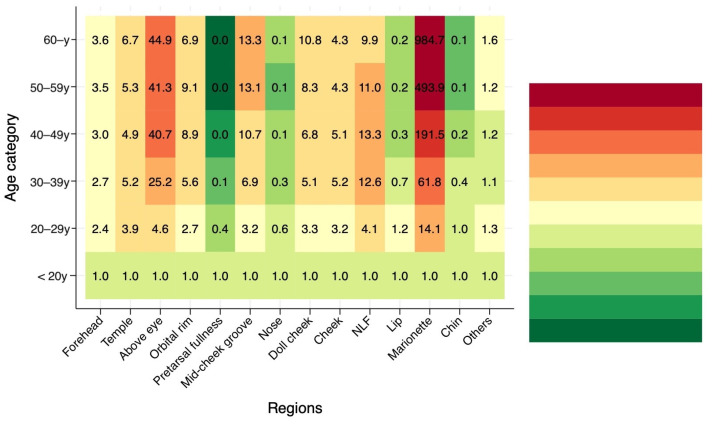
Odds ratio analysis of injection site selection by age group, using the “under 20 years” group as a reference (Reference = 1). The heatmap visualizes the relative likelihood of treating specific areas as age increases. The odds ratios for orbital rim and mid-cheek groove injections increase progressively, peaking in the 40s and 50s. Notably, the odds ratio for marionette line treatments shows a sharp, non-linear rise in patients over 40 (*p* < 0.05), indicating that this area becomes a distinct priority for mature patients.

In contrast, among patients aged 50–59 years, the treatment landscape shifted entirely toward rejuvenation; the orbital rim (30.1%) and nasolabial folds (26.8%) accounted for the majority of procedures, while requests for pretarsal fullness dropped to negligible levels. Furthermore, odds ratio analysis using the under-20 age group as a reference highlighted the increasing necessity of specific interventions with age (Figure 6).

The odds ratios for selecting the orbital rim and mid-cheek groove increased progressively with advancing age, peaking in the 40s and 50s. Notably, a sharp, non-linear rise in the odds ratio for marionette line treatments was observed from the 40s onwards (*p* < 0.05), indicating that this area becomes a specific and rapidly emerging concern in mature patients.

## 4. Discussion

This large-scale nationwide analysis demonstrates a definitive shift in the Japanese aesthetic market, where the rapid increase in hyaluronic acid procedures is driven primarily by middle-aged and older patients seeking rejuvenation rather than younger patients seeking augmentation. This demographic transition has fundamentally altered clinical practice, necessitating a move from single-site feature enhancement to multi-site structural restoration. The significant association observed between advancing age and the number of treated sites is rooted in the “Inside-Out” theory of facial aging, which posits that aging is a multifactorial process involving the volumetric deflation of deep fat compartments and the remodeling of the facial skeleton [9,10,11].

The trends observed in our Japanese cohort both align with and diverge from patterns documented in Western countries. The ASPS 2024 report confirms sustained growth in HA filler procedures in the United States (5.3 million in 2024, +1% from 2023), with middle-aged and older patients (≥40 years) representing the majority of recipients [1]. Similarly, the ISAPS 14-year global analysis documented a 40% increase in aesthetic procedures over the past four years, with HA fillers showing a 5.2% year-over-year increase [2]. These data corroborate our finding of robust market growth driven by mature demographics.

However, a notable divergence emerges when comparing age-specific trends. While our data demonstrate a clear shift toward older patients in Japan (with ≥40 years increasing from 39.4% in 2021 to 63.7% in 2024), European studies report concurrent growth among younger adults. Zarringam et al. documented that the proportion of patients aged 18–25 years seeking HA fillers in the Netherlands increased from 3.1% to 8.0% between 2008 and 2017 [3]. This suggests that Western markets may be experiencing bidirectional expansion—both younger individuals seeking preventative or enhancement treatments and older individuals seeking rejuvenation—whereas the Japanese market is characterized by a more pronounced unidirectional shift toward mature patients.

This divergence may reflect cultural differences in aesthetic attitudes and social media influence. Recent analyses have highlighted the role of Generation Z and millennials in driving younger-patient demand in Western countries, influenced by social media and celebrity culture [12,13]. In contrast, Japan’s rapidly aging society (with 29% of the population aged ≥ 65 years as of 2023) may exert a stronger demographic pressure on aesthetic medicine markets, prioritizing age-related concerns over youthful augmentation [14].

The shift toward orbital rim and mid-cheek treatments observed in our study aligns with anatomical characteristics specific to East Asian populations. As established by consensus statements, facial aesthetic goals in Asian populations are not aimed at “Westernization” but rather reflect distinct cultural beauty standards [6,7,15]. East Asian faces are characterized by greater midfacial retrusion, wider zygoma, and thicker skin compared to Caucasians [5,6]. These features render the midface particularly prone to flattening and volume loss with age, accentuating the nasolabial folds through soft tissue descent rather than simple wrinkling [16].

The marked rise in orbital rim and mid-cheek groove injections (reaching 30.1% in patients in their 50s) reflects a clinical response to the anatomical understanding of the aging orbit. The enlargement of the orbital aperture and resorption of the maxilla lead to a loss of bony support for the overlying soft tissues [17,18]. Studies by Wan et al. and Cotofana et al. have established that deep fat compartments (e.g., deep medial cheek fat) tend to undergo deflation with age, whereas superficial compartments (e.g., nasolabial fat) are more prone to inferior displacement [19,20]. The current trend toward mid-cheek and orbital rim injections suggests a paradigm shift from direct superficial filling to addressing the underlying structural cause by refilling deflated deep compartments, thereby providing anterior projection that indirectly repositions superficial fat pads [16].

In the lower face, our odds ratio analysis highlighted a sharp surge in marionette line treatments in patients over 40 (*p* < 0.05). This trend reflects the cumulative effects of mandibular bone resorption and weakening of the mandibular retaining ligaments [21,22]. Additionally, recent years have seen increased injections to the lateral face, including the temple. This reflects the adoption of modern “liquid lift” techniques that utilize fillers to reinforce the zygomatic and orbital retaining ligaments, generating a vector of lift that improves lower facial sagging—a comprehensive approach essential for older patients [23].

With the increasing complexity of multi-site treatments in anatomically dangerous zones such as the glabella and nasal region, safety remains paramount. In our previous analysis of this database, the incidence of serious complications, including vascular occlusion and infection, was 0.0041% (12 cases out of 299,413 sessions) [8]. All cases were managed with hyaluronidase, antibiotics, and/or corticosteroids, and were followed to treatment completion. This exceptionally low complication rate, despite the high volume of high-risk area treatments, can be attributed to rigorous standardization of injection techniques and strong emphasis on anatomical education within our clinic group. Recent expert consensus statements and evidence-based guidelines have reinforced the importance of standardized protocols and anatomical knowledge in minimizing adverse events [24,25,26].

This study has several important limitations that warrant consideration.

First and foremost, while our dataset encompasses 299,413 treatment sessions across 110 clinics nationwide, all data originated from a single large clinic group (Tokyo Chuo Beauty Clinic network). Although the clinics are geographically distributed across major cities in all prefectures of Japan, this single-network origin introduces potential selection bias. The observed trends may not fully represent the entire Japanese aesthetic medicine market, as they could be influenced by group-specific factors including: (a) Physician training and practice patterns: All practitioners within the network receive standardized training and follow unified clinical protocols, which may differ from practice patterns in independent clinics or other clinic chains; (b) Marketing strategies and patient demographics: The clinic group’s marketing approach, pricing structure, and brand positioning may attract a specific patient demographic that does not necessarily reflect the broader population seeking aesthetic treatments in Japan; (c) Geographic distribution and accessibility: Although the network covers all prefectures, the concentration of clinics in urban centers may underrepresent rural or suburban patient populations; (d) Referral patterns: The network’s reputation and specialization may lead to referral bias, with certain patient types or treatment complexities being overrepresented compared to the general market.

Despite these limitations, several factors support the validity and generalizability of our findings. The sheer scale of the dataset (nearly 300,000 sessions over 4 years) provides robust statistical power, and the nationwide geographic distribution mitigates regional bias. Furthermore, the demographic shift toward older patients and the evolution of injection site preferences align with broader societal trends in Japan, including population aging and evolving aesthetic ideals. Nevertheless, future multi-center studies incorporating data from diverse clinic groups—including independent practices and other chains—would be valuable to confirm the generalizability of these findings to the entire Japanese aesthetic market.

Second, the expansion of the clinic network from approximately 100 to 110 facilities during the study period inevitably contributed to the observed growth in patient volume. However, critical analysis reveals that clinic expansion alone cannot fully explain the trends. As shown in Table 1, the average number of patients per clinic increased dramatically from 391.7 in 2021 to 1036.4 in 2023 (+164.6%), despite clinic numbers growing only 56.3% during the same period. Notably, per-clinic patient volume continued to rise even after controlling for network expansion, suggesting genuine market growth rather than mere sampling artifact. Furthermore, the qualitative changes observed—specifically, the demographic shift toward older patients (aged ≥ 40 years comprising >50% of cases from 2023 onward) and the transition from single-site contouring to multi-site rejuvenation—represent fundamental shifts in treatment patterns that cannot be attributed to clinic expansion alone. These findings likely reflect broader societal trends in Japan, including population aging and evolving aesthetic preferences, rather than clinic-specific factors.

Third, the potential influence of product-specific factors warrants consideration. In 2021, the clinic network standardized its HA filler product line to YVOIRE (LG Chem, South Korea), which coincided with the implementation of a unified anatomical site classification system. To ensure data consistency, we excluded 2020 data from site-specific analyses. The question arises whether product-specific rheological properties influenced site selection patterns. However, several factors argue against this concern: (a) The YVOIRE portfolio offers a range of formulations with varying viscoelastic properties (Classic, Volume, Contour) suitable for diverse anatomical sites, from superficial to deep structural applications; (b) Our standardized protocol dictated that anatomical assessment and site selection preceded product choice, ensuring that clinical indications—not product availability—drove treatment decisions; (c) The observed demographic shift from younger patients seeking contouring to older patients requiring rejuvenation occurred progressively from 2021 to 2024, suggesting that evolving patient demand, rather than product characteristics, was the primary driver. Nevertheless, we acknowledge that product-specific training materials and marketing may have indirectly influenced physician practice patterns. Future multi-center studies incorporating diverse product portfolios would help validate the generalizability of our site-specific findings.

Fourth, as a retrospective database analysis, this study relies on accurate data entry and does not include patient-reported outcomes, satisfaction scores, or long-term follow-up data. While the standardized electronic medical record system minimized data entry errors, we cannot exclude the possibility of recording inaccuracies or omissions.

Fifth, due to database aggregation limitations, we were unable to extract precise year-by-year percentages for multi-site treatment sessions (≥2 sites) for inclusion in tabular format. Figure 3 illustrates the increasing trend, and we calculated that the mean number of sites per session rose from 1.32 in 2021 to 1.50 in 2024, suggesting an approximate increase in multi-site treatments from 32% to 40%. However, exact annual proportions require further data verification and are being obtained from our analytics provider.

Finally, the study did not distinguish between unique patients and repeat visitors. Each clinic visit was counted as a separate “patient” entry, meaning that individuals who returned for multiple treatments over the study period were counted multiple times. While this approach accurately reflects treatment session volume and clinical workload, it does not allow us to assess patient retention rates or the proportion of first-time versus returning patients. This limitation is inherent to the database structure and represents an area for improvement in future data collection systems.

Despite these limitations, the scale and geographic breadth of this dataset provide valuable insights into evolving trends in facial aesthetic medicine in Japan. The findings contribute to the growing body of evidence on age-related treatment patterns and underscore the importance of adapting clinical practice to the anatomical realities of aging populations.

## 5. Conclusions

This large-scale analysis of 417,590 injection sites across 299,413 treatment sessions confirms a paradigm shift in the Japanese aesthetic market: a transition from “contour augmentation” in younger patients to “structural rejuvenation” in middle-aged and older populations. The increasing prevalence of multi-site treatments—specifically targeting the orbital rim, mid-cheek, and marionette lines—reflects a clinical adaptation to the anatomical realities of skeletal resorption, deep fat deflation, and ligamentous laxity. As the demand for comprehensive facial rejuvenation continues to grow, practitioners must continue to refine their approaches based on a deep understanding of these age-related anatomical changes.

## Figures and Tables

**Table 1:** Annual Summary of Treatment Sessions, Injection Sites, and Patient Demographics. 2020 data cover October–December only (partial year). Data from 2020 were excluded from site-specific analyses due to non-standardized recording systems prior to 2021.

Year	Number of Clinics	Treatment Sessions	Injection Sites	Sites per Session	Patients per Clinics	Patients > 40 Years (%)
2020	-	2231	2597	1.16	-	-
2021	64	25,069	33,110	1.32	391.7	39.4
2022	76	50,335	67,559	1.34	662.0	45.2
2023	100	103,642	137,948	1.33	1036.4	60.0
2024	110	117,577	176,376	1.50	1068.9	63.7
Total	-	299,413	417,590	1.40	-	-

## Data Availability

The datasets presented in this article are not readily available because patient privacy and institutional data protection policies.

## Supplementary Materials

The following supporting information can be downloaded at: https://www.mdpi.com/article/10.3390/jcm15020893/s1, Table S1: Anatomical Definition and Clinical Indication of Injection Sites Used in the Standardized Classification System.
