# Supplementary material for: From Contouring to Rejuvenation: A Nationwide Big-Data Analysis of Hyaluronic Acid Injection Trends in Japan
Source: J Clin Med. 2026 Jan 22;15(2):893. doi: 10.3390/jcm15020893 (PMC12841990; doi:10.3390/jcm15020893)
Supplement: Supplementary file 1 [file jcm-15-00893-s001.zip › jcm-4088895-supplementary.pdf]

SUPPLEMENTARY MATERIAL

Supplementary Table S1. Anatomical Definition and Clinical Indication of Injection Sites Used in the Standardized Classification System.

Site	Anatomical Region/Boundary	Primary Aesthetic Indication	Notes
**Forehead**	Forehead region above the eyebrows to the hairline	Volume loss, frontal contour enhancement, correction of frontal bossing asymmetry	Targets frontal eminences or diffuse volume deficiency in the upper face
**Temple**	Lateral temporal region, including the temporal fossa and area superior to the zygomatic arch	Temporal hollowing, lateral facial lift effect	Often combined with lateral cheek treatment for comprehensive lifting
**Above eye**	Sub-brow region, specifically the retro-orbicularis oculi fat (ROOF) area	Correction of upper eyelid hollowing, enhancement of brow fullness	Addresses deepening of the superior sulcus and sunken upper eyelid appearance
**Orbital rim**	Inferior orbital rim and tear trough region, addressing the palpebromalar groove (lid-cheek junction)	Tear trough deformity, lid-cheek junction depression, periorbital rejuvenation	Includes both medial tear trough and lateral orbital rim extension
**Pretarsal fullness**	Subcutaneous pretarsal orbicularis oculi muscle region of the lower eyelid	Creation of lower eyelid fullness (aegyo-sal) for youthful appearance	Popular aesthetic feature in East Asian populations; creates convex lower lid contour
**Mid-cheek groove**	Midface depression extending obliquely from the medial canthus inferolaterally (nasojugal groove)	Correction of midface depression, "Golgo line" or nasojugal groove	Often reflects deflation of deep medial cheek fat and/or maxillary retrusion
**Nose**	Nasal dorsum, tip, columella, or alar base	Dorsal augmentation, tip refinement, correction of asymmetry or post-rhinoplasty irregularities	Non-surgical rhinoplasty; includes bridge augmentation and tip projection
**Doll cheek**	Anterior malar prominence, creating anterior projection of the cheek	Fuller, more anterior cheek contour; "apple cheek" appearance	Emphasizes anterior rather than lateral cheek volume
**Cheek**	Lateral and posterior malar region, lateral zygomatic area, and submalar hollow	Lateral cheek augmentation, correction of submalar concavity, midface volumization	Provides structural support for lateral facial contour and midface lift
**NLF (Nasolabial fold)**	Groove extending from the lateral alar base to the oral commissure	Softening of nasolabial fold depth, midface rejuvenation	Can be treated directly (superficial) or indirectly (deep structural support)
**Lip**	Upper lip, lower lip, vermillion border, or philtral columns	Lip volume enhancement, border definition, correction of asymmetry or age-related volume loss	Includes vermillion augmentation, cupid's bow definition, and perioral contouring
**Marionette line**	Depressed groove extending inferiorly from the oral commissure toward the jawline (melomental fold)	Correction of oral commissure descent, lower face rejuvenation	Becomes prominent with age due to mandibular resorption and ligamentous laxity

**Chin**	Mental protuberance, pre-jowl sulcus, and anterior mandible	Chin projection, jawline definition, correction of chin ptosis or pre-jowl depression	Includes mentoplasty effect and jawline contouring
**Others**	Sites not categorized above	Miscellaneous indications	Includes mandibular angle, jawline, neck, and other less common regions

**Abbreviations: ** ROOF, retro-orbicularis oculi fat; NLF, nasolabial fold.

Note: Each anatomical site was selectable via a standardized dropdown menu in the electronic medical record system. Free-text entry was not permitted, ensuring consistent classification across all clinics. The treating physician selected the appropriate site(s) immediately after each procedure, with multiple selections allowed for multi-site treatments.
